# Heterocyclic Molecular Targeted Drugs and Nanomedicines for Cancer: Recent Advances and Challenges

**DOI:** 10.3390/pharmaceutics15061706

**Published:** 2023-06-10

**Authors:** Junxia Liu, Tengfei Chao, Yingying Liu, Chen Gong, Yinan Zhang, Huihua Xiong

**Affiliations:** 1 Department of Oncology, Tongji Hospital, Tongji Medical College, Huazhong University of Science and Technology, Wuhan 430000, China; d202282220@hust.edu.cn (J.L.); tengfeichao@tjh.tjmu.edu.cn (T.C.); chengong@tjh.tjmu.edu.cn (C.G.); 2 School of Chemistry and Chemical Engineering, Frontiers Science Center for Transformative Molecules and National Center for Translational Medicine, Shanghai Jiao Tong University, Shanghai 200000, China; yangyang21@sjtu.edu.cn; 3 School of Chemical Science and Engineering, Tongji University, Shanghai 200000, China

**Keywords:** heterocyclic compounds, molecular targeted drugs, nanomedicine, cancer

## Abstract

Cancer is a top global public health concern. At present, molecular targeted therapy has emerged as one of the main therapies for cancer, with high efficacy and safety. The medical world continues to struggle with the development of efficient, extremely selective, and low-toxicity anticancer medications. Heterocyclic scaffolds based on the molecular structure of tumor therapeutic targets are widely used in anticancer drug design. In addition, a revolution in medicine has been brought on by the quick advancement of nanotechnology. Many nanomedicines have taken targeted cancer therapy to a new level. In this review, we highlight heterocyclic molecular-targeted drugs as well as heterocyclic-associated nanomedicines in cancer.

## 1. Introduction

Cancer is one of the most common causes of death, and it imposes a heavy economic and medical burden on society as a whole. With the development of molecular biology and cytogenetics, the molecular mechanism of tumorigenesis and development have been found to be highly complex, involving chromosomal abnormalities, oncogene amplification, deletion of tumor suppressor genes, up-regulation of growth factors and their receptors, and activation of tumor-related signal transduction pathways, etc. [1,2,3]. In order to effectively treat cancers in patients, researchers seek novel anticancer medications with high selectivity, minimal side effects, and even the ability to overcome drug resistance. Anticancer drug research and development have now advanced beyond cytotoxic agents to targeted drugs and nanomedicines [4]. The anticancer effects of targeted drugs and nanomedicines can be mediated by many pathways, resulting in remarkable outcomes [5,6,7,8,9].

Heterocyclic compounds, which are made up of both carbon and non-carbon atoms, serve as a crucial structural foundation for numerous chemicals with pharmacological and biological value. The research on heterocyclic compounds is an important part of organic chemistry and is utilized extensively in many industries, especially medicine [10,11,12,13]. Currently, heterocyclic compounds serve as the main active ingredient in a variety of pharmaceuticals, including analgesics, anti-inflammatory drugs, anti-tubercular drugs, antihypertensives, antidepressants, and even anticancer drugs [14,15,16,17]. In recent decades, many novel heterocyclic targeted drugs have emerged.

Nanomedicine is a relatively new area of medical study. It involves the use of nanotechnology to address medical issues and has tremendous potential for precision medicine [18,19,20]. The application of nanomedicine to the diagnosis and treatment of a wide range of illnesses, including cardiovascular diseases, respiratory diseases, infectious diseases, and Alzheimer’s disease, has improved patient outcomes [21,22,23,24,25]. More significantly, the advancement of precision medicine in cancer has been aided by the advent of nanomedicine [26,27,28,29]. Nanotechnology has improved the sensitivity and specificity of current diagnostic methods for tumor biomarkers and even imaging detection [30,31,32,33,34]. Existing anticancer medications have many limitations that can be addressed using nanotechnology, including significant side effects, limited water solubility, and low absorption. Nanotechnology can even combine multiple drugs and deliver them to target sites to exert anti-tumor effects. Furthermore, the development of many nanomedicines and nanomedicine delivery systems has pushed the targeted therapy of cancer to new levels. Heterocyclic-associated nanomedicines make up a large proportion. In this review, we highlight heterocyclic molecular targeted drugs and heterocyclic-associated nanomedicines in cancer.

## 2. New Potential Heterocyclic Molecular Targeted Drugs

**Figure 1:**
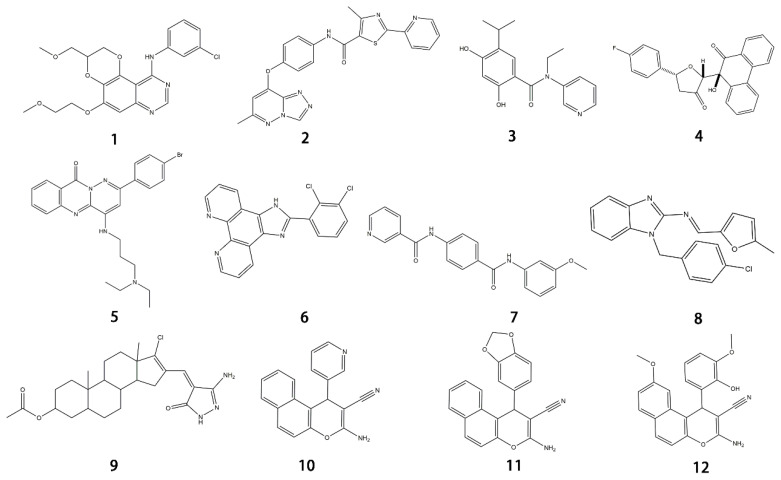
Chemical structure formulas for compounds **1**–**12**.

**Figure 2:**
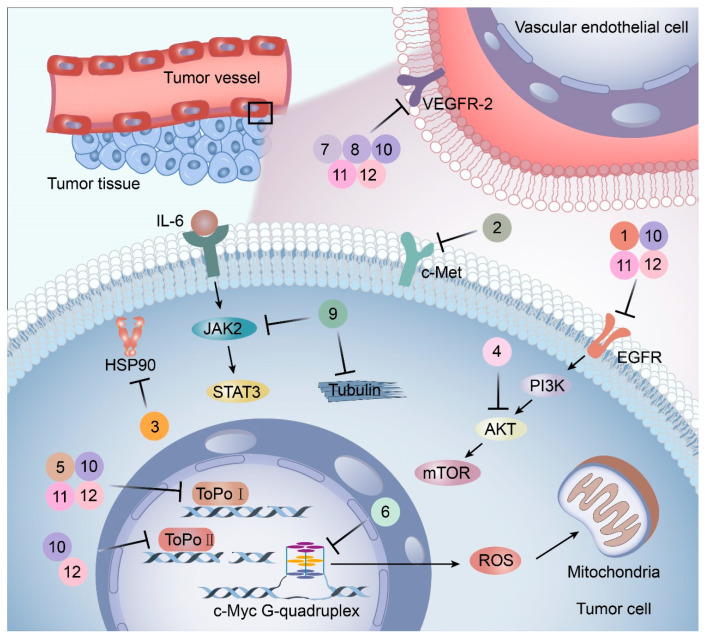
Targets and mechanisms of new potential heterocyclic targeted drugs. The targets and mechanisms of new potential heterocyclic molecular targeted drugs 1–12 for cancer were reviewed based on the spatial localization of therapeutic targets in tumor cells.

Molecular targeted therapy is a key element of the new era of comprehensive multidisciplinary cancer treatment [35,36,37]. A considerable number of molecular targeted medications have been created as part of the development of targeted therapy. We describe new potential heterocyclic molecular targeted drugs based on the spatial localization of therapeutic targets in tumor cells (Figure 1 and Figure 2, Table 1 and Table 2).

### 2.1. Drugs Acting on Cell Membranes

EGFR is widely distributed on cell surfaces and participates in modulating cell growth, proliferation, and differentiation [38]. A set of heterocyclic 2,3-dihydro-[1,4]dioxino [2,3-f] quinazoline derivatives to target EGFR were conceived and created by Qin et al. IC50 is the concentration of a drug that inhibits 50 percent of targets. All substances were shown to have the ability to inhibit EGFR kinase according to the enzyme assay results (IC_50_ = 10.29–652.3 nM). Compared with erlotinib and gefitinib, compound **1** showed the strongest inhibition of EGFR kinase, with an IC_50_ value of 10.29 nM. Anti-proliferation showed that compound **1** could inhibit the proliferation of A549 and NCI-H157 cell lines. The median cytotoxic concentration (CC50) is the concentration of a drug that causes 50% of normal cells to die. The larger difference between CC50 and IC50 values of tumor cells or between the IC50 values of normal and tumor cells, the safer and less toxic the drug. The CC50 of compound **1** against the human renal epithelial cell line T293 was larger than 100 μM, and the CC50 against the normal lung cell line WI-38 was 90.55 μM, indicating that compound **1** had no significant cytotoxic effect in vitro. Further studies demonstrated that compound **1** was joined to the ATP-binding pocket of the EGFR active site [39].

It has been reported that c-Met targeting can be used in the treatment of NSCLC, prostate cancer, gastric cancer, and other tumors [40,41,42,43]. New triazolo-pyridazine/pyrimidine derivatives were synthesized, evaluated, and found to inhibit c-Met kinase in A549, MCF-7, and HeLa cell lines that overexpressed c-Met. Among them, compound **2**, the most effective compound, displayed obvious cytotoxicity to A549, MCF-7, and HeLa cell lines, with IC_50_ values of 1.06 ± 0.16, 1.23 ± 0.18, and 2.73 ± 0.33 μM, respectively. Meanwhile, compound **2** significantly inhibited c-Met kinase activity, with an IC_50_ value of 0.090 μM, similar to Foretinib. Acridine orange staining and flow cytometry analysis revealed that compound **2** could not only boost apoptosis in A549 cells but also trigger G0/G1 phase cell cycle arrest. Mechanically, the 5-methyl thiazole fragment is introduced into the five-atom fraction, which is essential for c-Met inhibition. Compound **2** could be a prospective class II c-Met inhibitor [44].

### 2.2. Drugs Acting on the Cytoplasm

A protein-folding chaperone known as the heat shock protein (HSP) maintains the stability of numerous signal transduction proteins in cells to assist in cell growth and survival [45]. HSP90 inhibitors, a group of ring-opening dihydroxy benzamide compounds, were devised and synthesized by Liu et al. Compound **3** showed the obvious inhibition of HSP90, with an IC_50_ value of 110.18 nM as well as BIIB021, and led to the degradation of AKT downstream signaling, which was concentration- and time-dependent. The GI_50_ values of compound **3** were 0.07 μM against the KRAS mutant A549 cell line and 0.05 μM against the EGFR mutant H1975 cell line. Compound **3** had stronger inhibition of cell proliferation and metastasis than 17-AAG and BIIB021. Compound **3** prevented A549 cells from migrating, with an IC_50_ value of 1 μM. Compound **3** is a highly permeable drug with little toxic potency or cardiotoxicity. Pharmacokinetic tests showed that its oral bioavailability was 17.8%. Additionally, compound **3** exhibited anti-tumor activity at a daily dose of 50 mg/kg, with 72% tumor growth delay in a nude mouse A549 lung xenograft model. In the lung H1975 xenograft model, the combination therapy of compound **3** and afatinib resulted in a 67.5% tumor growth inhibition. Therefore, compound **3** is a promising potential lung cancer treatment medication [46].

The dysfunction of the PI3K-AKT-mTOR signaling pathway can not only lead to neurodegeneration, cancer, and other diseases but also induce drug resistance [47,48]. Huang and colleagues created a series of dihydrofuran-3-one and 9, 10-phenquinone hybrid compounds. CCK-8 assay showed that these compounds had inhibitory effects on HCT-116, A549, and SJSA-1 cells, and the IC_50_ value of HCT-116 cells was between 0.92 and 3.91 μM. Compound **4** was the most cytotoxic to HCT-116 cells and could arrest the cell cycle in the G2/M phase. Molecular docking assays revealed that compound **4** had a more significant inhibitory effect on AKT kinase than CAL-101. Compound **4** exerts its anti-tumor effects as an AKT kinase inhibitor by competitive binding to ATP to limit intradomain and interdomain motility. This result was further confirmed by a kinase selectivity assay [49].

### 2.3. Drugs Acting on the Cell Nucleus

DNA topoisomerase (Topo), an enzyme in the nucleus, is involved in DNA replication, recombination, transcription, etc. Huang et al. designed and synthesized more than 30 pyridazino[1,6-b]quinazolinones derivatives. Compared with CPT and doxorubicin positive controls, compound **5** caused clear moderate or even potent cytotoxicity against the SK-OV-3, CNE-2, MGC-803, NCI-H460, and LO-2 cell lines (IC50 = 1.93 ± 0.16, 2.33 ± 0.52, 1.39 ± 0.14, 1.55 ± 0.14, 5.71 ± 0.60, respectively). Further studies revealed that most compounds could be embedded in DNA molecules. The Topo I inhibition assay indicated that Topo I was severely inhibited by compound **5**. In a dose-dependent manner, compound **5** elicited mild G2 cell cycle arrest and apoptosis. In the MGC-803 xenograft tumor model, compound **5** had a significant inhibitory effect, with total growth inhibition reaching 55.9%. Additionally, molecular docking studies demonstrated that compound **5** might bind to Topo I and reduce its activity compared to doxorubicin. In conclusion, pyridazino[1,6-b]quinazolinones are novel drug scaffolds that can be used to produce anticancer medications [50].

G-quadruplex (G4s) plays a significant role in biological activities by regulating telomerase function, gene expression, and the composition of topologically associated domains [51,52]. Using microwave-assisted technology, Wu et al. designed and synthesized a range of phenomidazole derivatives containing phenomidazole and imidazole heterocyclic aromatic rings, which are new drugs with anti-tumor activity. In comparison to doxorubicin, compound **6** inhibited nasopharyngeal carcinoma CNE-1 cell proliferation in a dose-dependent manner, with an IC_50_ value of 1.1 μM. Compound **6** showed low toxicity to human keratinocyte HaCaT cells, with an IC_50_ value of 16.8 μM. It could also induce apoptosis and G0/G1 cycle arrest in CNE-1 cells. Mechanistically, compound **6** could exert anti-tumor effects by stabilizing c-Myc G4s DNA, leading to DNA damage and thereby activating reactive oxygen species-mediated mitochondrial dysfunction. In the zebrafish xenograft model, compound **6** significantly inhibited the growth of xenograft tumors and had only a low toxic effect on zebrafish embryo development under the precondition of an effective concentration [53].

**Table 1:** New potential molecular targeted heterocyclic compound drugs.

The Spatial Location of Target	Compound Name	Chemical Structure Formula	Targets [IC50 (μM)]	Cancer	Cell [IC/GI/CC50 (μM)]	Reference
Cell membrane	**1**	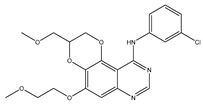	EGFR [IC50 = 10.29 × 10^−3^]	NSCLC	A549 [IC50 = 9.95]	[39]
					NCI-H157 [IC50 = 11.66]	
					T293 [CC50 > 100]	
					WI-38 [CC50 = 90.55]	
	**2**	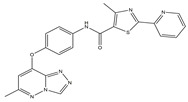	c-Met [IC50 = 90.00 × 10^−3^]	Lung, liver and breast cancers	A549 [IC50 = 1.06 ± 0.16]	[44]
					MCF-7 [IC50 = 1.23 ± 0.18]	
					HeLa [IC50 = 2.73 ± 0.33]	
					LO2 [IC50 > 50.00]	
Cytoplasm	**3**	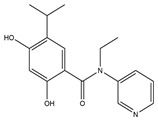	HSP90 [IC50 = 110.18 × 10^−3^]	Lung cancer	A549 [GI50 = 0.07 ± 0.01]	[46]
					H1975 [GI50 = 0.05 ± 0.01]	
					Hep3B [GI50 = 0.20 ± 0.03]	
					MDA-MB-231 [GI50 = 0.09 ± 0.01]	
	**4**	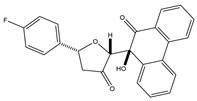	AKT [NA]	Colon cancer	HCT-116 [IC50 = 0.92 ± 0.05]	[49]
Cell nucleus	**5**	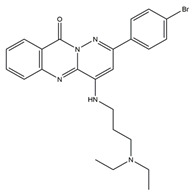	Topo I [NA]	Ovarian, nasopharyngeal, stomach and lung cancers	SK-OV-3 [IC50 = 1.93 ± 0.16]	[50]
					CNE-2 [IC50 = 2.33 ± 0.52]	
					MGC-803 [IC50 = 1.39 ± 0.14]	
					NCI-H460 [IC50 = 1.55 ± 0.14]	
					LO-2 [IC50 = 5.71 ± 0.60]	
	**6**	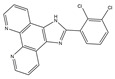	c-Myc G-quadruplex [NA]	Nasopharyngeal cancer	CEN-1 [IC50 = 1.1 ± 0.1]	[53]
					HaCaT [IC50 = 16.8 ± 0.7]	
TME	**7**	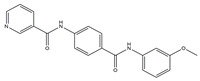	VEGFR-2 [IC50 = 2.17]	Breast, liver and colon cancers	MCF-7 [IC50 = 1.37]	[54]
					HepG-2 [IC50 = 1.05]	
					HCT-116 [IC50 = 1.46]	
					WI-38 [IC50 = 60.8]	
	**8**	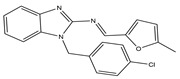	VEGFR-2 [NA]	Lung metastasis of melanoma	NA	[55]
Multiple targets	**9**	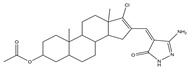	JAK2/Tubulin [NA]	Lung cancer	A549 [IC50 = 27.36]	[56]
	**10**–**12**	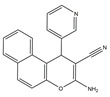	Topo I/Topo II/EGFR/VEGFR-2 [IC50 = 0.1392–0.6349]	Breast, liver and colon cancers	MCF-7 [IC50 = 1.6–2.2]	[57]
		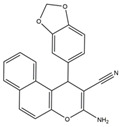			HLF-1 [IC50 = 19.1–24.3]	
		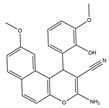			WI-38 [IC50 = 20.2–26.5]	

NA: Not applicable.

**Table 2:** The name of compound **1**–**12**.

Compound	Name
**1**	N-(3-chlorophenyl)-5-(2-methoxyethoxy)-3-(methoxy-methyl)- 2,3-dihydro-[1,4]dioxino[2,3-f]quinazolin-10-amine
**2**	4-Methyl-N-(4-((6-methyl-[1,2,4]triazolo[4,3-b]- pyridazin-8-yl)oxy)phenyl)-2-(pyridin-2-yl)thiazole-5-carboxamide
**3**	N-Ethyl-2,4-dihydroxy-5-isopropyl-N-(pyridin-3-yl)benzamide
**4**	(2R,5R)-5-(4-Fluorophenyl)-2-((S)-9-hydroxy-10-oxo-9,10-dihydrophenanthren-9- yl)dihydrofuran-3(2H)-one
**5**	2-(4-Bromophenyl)-4-((3-(diethylamino)propyl)amino)-10Hpyridazino [6,1-b] quinazolin-10-one
**6**	2-(2,3-dichlorophenyl)-1H-imidazo[4,5-f][1,10]phenanthroline
**7**	N-(4-((3-Methoxyphenyl)carbamoyl)phenyl)nicotinamide
**8**	1-(4-chlorobenzyl)-2-(5-methyl-2-furfurylidenenamino)-benzimidazole
**9**	(Z)-16-((3-amino-5-oxo-1,5-dihydro-4H-pyrazol-4-ylidene)methyl)-17-chloro-10,13-dimethyl-2,3,4,5,6,7,8,9,10,11,12,13,14,15-tetradecahydro-1H-cyclopenta[a]phenanthren-3-yl acetate
**10**	3-Amino-1-(pyridin-3-yl)-1H-benzo[f]chromene-2-carbonitrile
**11**	3-Amino-1-(benzo[d][1,3]dioxol-5-yl)-1H-benzo[f]chromene-2- carbonitrile
**12**	3-Amino-9-methoxy-1-(2-hydroxy-3-methoxyphenyl)-1H-benzo [f]-chromene-2-carbonitrile

### 2.4. Drugs Acting on the Tumor Microenvironment (TME)

The surrounding milieu of tumor cells is called the tumor microenvironment. It is made up of the extracellular matrix, bone marrow-derived inflammatory cells, fibroblasts, immune cells, and adjacent blood vessels. Using sorafenib as the lead compound, Ran et al. designed and synthesized three series of novel compounds containing pyridine and evaluated these compounds for VEGFR-2 suppression and anticancer activity with Sorafenib as the positive control. Compared with the control group, compound **7** exhibited significant cytotoxicity effects on MCF-7, HepG-2, HCT-116, and WI-38 cell lines (IC50 of 1.37, 1.05, 1.46, and 60.8 μM, respectively).. More importantly, compound **7** had the strongest inhibitory effect on VEGFR-2 (IC_50_ = 2.17 μM). In contrast to the untreated group, further research revealed that compound **7** considerably up-regulated the expressions of caspase 3 and Bax while drastically downregulating the expression of Bcl-2 in HepG-2 cells. Compound **7** might encourage apoptosis and cause G2/M cell cycle arrest in HepG-2 cells according to flow cytometry studies. Additionally, when compared to celecoxib (87%), compound **7** considerably reduced the level of tumor necrosis factor α expression. At the same time, it also restrained the proliferation of vascular smooth muscle cells in a concentration-dependent manner. The binding of the compound to the ATP pocket of VEGFR-2 was found by the molecular docking method, which provided a basis for further elucidation of the structure–activity relationship [54].

Similarly, compound **8** (MFB), a small molecule compound containing two amino benzimidazoles designed and synthesized by Hsu et al., might target VEGF/VEGFR to prevent angiogenesis and lymphangiogenesis. By blocking VEGF-A and VEGF-C signaling, compound **8** prevented human umbilical vein endothelial cells and lymphatic endothelial cells from proliferating, migrating, invading, and forming endothelial tubes in vitro. The results were also confirmed in animal experiments. Additionally, a mouse model of lung metastasis of melanoma confirmed that compound **8** showed an anti-tumor metastasis effect in vivo. Molecular docking experiments revealed that binding to VEGFR-2 might be the mechanism of compound **8** in inhibiting angiogenesis and lymphangiogenesis [55].

### 2.5. Drugs Acting on Multiple Targets

The importance of the JAK2/STAT3 signaling pathway in numerous disorders has been established by researchers. With the use of the A549 cell line as the experimental model, six 3′-acetoxy-5′-androsterane heterocyclic compounds were chosen for testing their antiproliferative effects on NSCLC. Compound **9** was the most toxic to A549 cells (IC_50_ = 27.36 μM). According to the findings of molecular docking studies, the anticancer activity of compound **9** might be caused by its strong affinity for JAK2 and tubulin-colchicine soblidotin. The tubulin assay further verified the activity of compound **9** in inhibiting tubulin polymerization. According to flow cytometry research, compound **9** could not only accelerate the apoptosis of A549 cells but also halt the progression of the cell cycle, causing pre-G1 and G2/M cell cycle arrest. Additionally, compound **9** induced DNA fragmentation, up-regulation of apoptotic genes, down-regulation of anti-apoptotic genes, and mitochondrial dysfunction. Therefore, compound **9** is a multitarget anticancer drug that has great application potential [56].

El-mawgoud et al. designed and synthesized novel rigid analogs of 2-naphtho and studied their biological activities. When tested on the breast cancer cell line MCF-7, colon cancer cell line HCT-116, and liver cancer cell line HepG-2, compounds **10**–**12** showed inhibitory activity comparable to vinblastine and doxorubicin. Compounds **10** and **11** demonstrated the highest levels of cytotoxicity against MCF-7, HCT-116, and HepG-2 cell lines (IC_50_ = 2.2, 2.4, and 2.4 μM, respectively, for compound **10,** and 1.6, 1.5, and 5.5 μM, respectively, for compound **11**). With an IC_50_ value of 2.1 μM, compound **12** also demonstrated strong cytotoxicity against the breast cancer cell line MCF-7. In comparison to the control group, treatment groups with compounds **10**, **11,** or **12** increased MCF-7 cell apoptosis and G2/M cell cycle arrest. Mechanistically, they exerted strong anticancer effects by targeting Topo I and II, EGFR, and VEGFR-2 (IC50 = 0.1392–0.6349 μM). Compounds **10**–**12** showed weak inhibitory activity against HFL-1 and WI-38 cells with IC50 values ranging from 19.1 to 26.5 μM. They were also characterized by good oral bioavailability and high capacity for transport. Together, these compounds have great potential for cancer therapy [57].

## 3. Heterocyclic-Associated Nanomedicines

**Figure 3:**
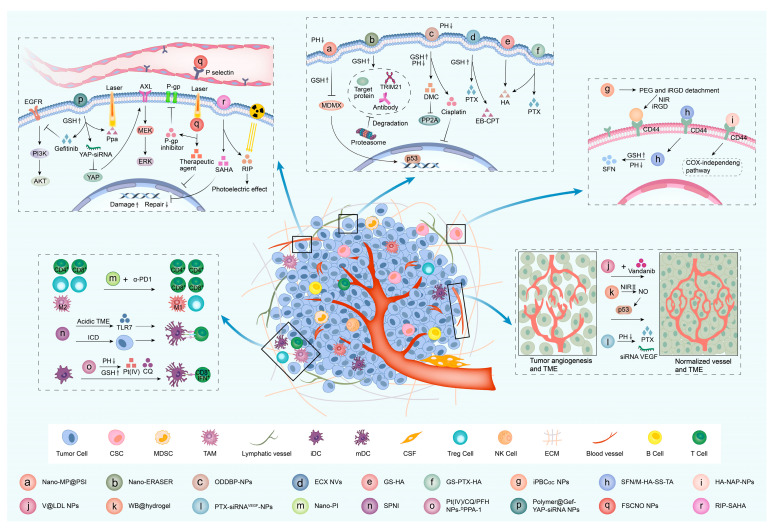
Effects and mechanisms of heterocyclic-associated nanomedicines. The mechanisms of heterocyclic-related targeted nanomedicines for cancer were reviewed based on their effects, including inhibiting tumor cells, inhibiting CSCs, inhibiting tumor angiogenesis, regulating the immune microenvironment, and reversing drug resistance.

The application of nanotechnology in drug development can not only reduce the side effects of drugs but also improve the water solubility of drugs and the concentration of drug accumulation in tumors. The development of nanomedicines has dramatically advanced the targeted therapy of cancer. Heterocyclic-related targeted nanomedicines account for a large proportion of nanomedicines. We classified heterocyclic-associated nanomedicines according to their effects (Figure 3, Table 3).

### 3.1. Inhibiting Tumor Cells

As is well known, the MDMX protein, which is a disorder of gene expression in a variety of malignancies and suppresses the p53 protein, has received much attention and has been developed as a possible therapeutic target for cancer [58,59,60]. Yan et al. designed and synthesized a supramolecular gold (I) -thiol-peptide complex (Nano-MP). They also manufactured polyacryl sulfydryl imidazole (PSI) to modify Nano-MP in order to enhance targeting, as Nano-MP is sensitive to the pH of TME. Nano-MP@PSI can release cargo through the glutathione (GSH) trigger. Nano-MP@PSI had longer blood-circulation time and greater tumor-specific accumulation compared to Nano-MP. Further experiments revealed that Nano-MP@PSI could be excreted in a mononuclear phagocytic system-dependent manner. Mechanism experiments indicated that Nano-MP@PSI may target the P53 and P73 pathways by degrading MDMX proteins to treat tumors. Furthermore, it was demonstrated that Nano-MP@PSI inhibited tumor growth by degrading MDMX protein in mouse xenograft models of retinoblastoma and pancreatic cancer [61]. Similarly, Nano-ERASER, a nanogel system with the ability to bind, transport, and release antibodies, was reported by Sui and colleagues. Nano-ERASER, carrying antibodies into the tumor cells, released antibodies through stimulation by high concentrations of GSH. The antibody bound to the target protein and TRIM21 to form a complex. The proteasome then degraded important proteins in cancer cells and performed an anti-tumor function [62].

Additionally, nanotechnology can facilitate the delivery of conventional chemotherapy medications to their intended areas, increasing treatment effectiveness and serving a similar function in tumor suppression as targeted therapies. Yang’s team created precisely pH-regulated dual-drug-backboned nano-prodrug ODDBP-NPs to address the drastic differences in pH values in the blood, TME, and tumor intracellular environment. The ODDBP-NPs can maintain high stability in the blood, exhibit DePEGylation and size changes in the TME, and stimulate the release of cisplatin and Demethyl-cantharidin (DMC) in the acidic and high GSH intracellular environment. DMC can significantly inhibit PP2A to have an anti-tumor effect. According to subsequent cell and animal experiments, the ODDBP-NPs can increase the concentration of tumor drug accumulation and the absorption of tumor cells to exert a greater anti-tumor function [63]. Zhou et al. reported that paclitaxel-mediated co-assembly with a camptothecin predrug produced vesicular nanomedicine ECX NVs with a drug loading efficiency close to 100%. Compared with CPT, the effect of an EB-CPT:PTX mass ratio of 1:2 on HCT116 cells was 50%, and the combination index was 0.59. More importantly, EB-CPT:-PTX significantly improved the anti-tumor effect in vivo [64]. Biocompatible copolymer Soluplus^®^ was surface modified with glucose and then loaded with histamine (HA) and paclitaxel (PTX) to form multiple co-loaded micellar nanodelivery systems. Further studies clarified that GS-HA and GS-PTX-HA had better anti-breast-cancer effects in vitro and vivo. These results show that GS-HA and GS-PTX-HA have broad prospects in breast cancer treatment [65].

### 3.2. Inhibiting Cancer Stem Cells (CSCs)

Numerous heterocyclic-related nanomedicine systems have been discovered that target cancer stem cells in addition to common tumor cells. There is a small subset of tumor cells that have the ability to self-renew and generate heterogeneous tumor cells called CSCs [66,67]. CSCs are thought to be fundamental in the development, spread, and recurrence of cancer [68]. Zhu et al. designed and developed a cascade-responsive nano-assembly system of iPBC_GC_ NPs consisting of the HSP inhibitor gambogic acid, CR-based organic photothermal agents, and a targeted vector. The nano-assembly system is used in conjunction with photochemotherapy to target CD44+ CSCs to effectively inhibit tumor progression and metastasis [69].

Sulforaphane (SFN), a naturally anticancer isothiocyanate found in broccoli, has been used to treat malignancies of the breast, prostate, pancreas, and other organs [70,71,72]. According to various studies, SFN can treat breast cancer by preventing the production of breast cancer stem cells (BCSCs) [73,74]. Gu et al. reported a nano-carrier M-HA-SS-MA that can load SFN and improve its high hydrophobicity and instability. Higher packaging and loading efficiencies for SFN were achieved by M-HA-SS-MA, at 92.3 ± 62.17% and 33.64 ± 1.33%, respectively. M-HA-SS-MA further evaluated the GSH and pH-responsive effects of the nanocarrier and found that the nanocarrier can maintain greater stability in the blood circulation system and can separate and release drugs in the reductive and weakly acidic environment of the tumor. Subsequent experiments revealed that SFN/M-HA-SS-TA targeted CD44-positive breast cancer cells and BCSCs. In vivo experiments further confirmed this result and showed that SFN/M-HA-SS-TA had good biosafety [75]. To specifically target BCSCs, hyaluronic acid-coated naproxen nanoparticles (HA-NAP-NPs) were created. In addition to better controlling NAP release and enhancing the blood compatibility of biomaterials, HA coatings can interact with CD44+ to hasten CSC drug uptake and boost the effectiveness of anti-CSCs. Mechanistically, HA-NAP-NPs are a potential treatment for breast cancer through the cox-independent pathway to target BCSCs [76].

### 3.3. Inhibiting Tumor Angiogenesis

Tumor angiogenesis, which is regulated by angiogenic and anti-angiogenic factors, is the process by which a tumor develops new blood vessels on the basis of primary microvessels [77]. In addition to providing nutrients for tumor growth and metabolic pathways, tumor neovascularization creates favorable conditions for tumor cell metastasis [78,79]. As a result, anti-angiogenesis therapy, which can restrain tumors by inhibiting tumor angiogenesis and normalizing tumor blood vessels, has become one of the key elements of tumor therapy [80,81,82]. Vandetanib, a small-molecule tyrosine inhibitor targeting vascular endothelial growth factor receptor (VEGF) and epidermal growth factor receptor (EGFR) as well as RET kinase, is commonly used to treat locally advanced or metastatic inoperable medullary thyroid carcinoma [83,84,85]. V@LDL NPs are nano-delivery carriers that can carry and constantly release Vandetanib. In vitro and in vivo studies demonstrated that the combination of V@LDL NPs with Vandetanib can continuously suppress tumor angiogenesis and normalize tumor vessels, lasting for at least five days. Moreover, V@LDL NPs and Vandetanib may inhibit and normalize tumor blood vessels to further reprogram the tumor immune microenvironment and impede tumor growth and metastasis [86].

Nitric oxide (NO) is a key regulator of physiological processes such as vasodilation, nerve conduction, digestion, and gonadal hormone modulation [87,88,89]. Many investigations have revealed that NO can promote p53 activation and accumulation in a variety of illnesses, including cancer [90,91,92,93]. WB@hydrogel, an NIR-II responsive anti-angiogenic nanomedicine, is different from traditional angiogenic inhibitors. In response to NIR-II, the drug can produce and release NO to promote the expression of the wild-type p53 protein and then reverse the tumor pro-angiogenic microenvironment into an anti-angiogenic microenvironment in which the expression of angiogenic factors decreases and the expression of angiogenesis inhibitors increases [94]. Jin’s team prepared a double-loaded nano drug delivery system to achieve the co-loading of PTX and siRNA^VEGF^ (PTX-siRNA^VEGF^-NPs), which enhanced the anti-tumor effect through the direct tumor inhibition of PTX, and the anti-angiogenesis effect of siRNA^VEGF^. PTX-siRNA^VEGF^-NPs are characterized by a particle size of 85.25 nm, a zeta potential of 5.25 mV, and good plasma stability. The findings of an in vitro experiment demonstrated that PTX-siRNA^VEGF^-NPs can greatly reduce the level of VEGF mRNA expression, suppress the proliferation of breast cancer 4T1 cells, and increase the apoptosis of breast cancer 4T1 cells. A 4T1 breast cancer mouse in situ model was used to study the anti-tumor and anti-angiogenic effects of PTX-siRNA^VEGF^-NPs in vivo. The results of animal experiments indicated that the anti-tumor effect of the PTX-siRNA^VEGF^-NP group was superior to that of the PTX-NP and siRNA^VEGF^-NP groups. The detection of VEGF mRNA and VEGF expression levels in mouse tumor tissues revealed that the expression level of the PTX-siRNA^VEGF^-NP group was the lowest. In order to determine whether PTX-siRNA^VEGF^-NPs were safe, the body weight of mice and their serum levels of ALT, AST, IFN- and IL-6 were measured after drug administration. It was discovered that neither the body weight nor any other indicators significantly changed, suggesting PTX-siRNA^VEGF^-NPs are safe [95].

### 3.4. Regulating the Immune Microenvironment

The cancer immunosurveillance hypothesis, which states that the body’s immune system might serve a monitoring function to identify and eliminate malignant cells, was first put forth by Burnet and Thomas [96,97]. Through a variety of mechanisms, tumor cells accomplish their immunological escape from detection and attack by the body’s immune system, which leads to the formation of tumors. Through the use of immunomodulators, antibodies, immune cells, etc., tumor immunotherapy utilizes a new anti-tumor therapy based on the body's built-in defense system [98]. These immunotherapy approaches trigger the body’s immune system and immune cells to play an anti-tumor role. Nanomedicine can improve the accumulation of immunomodulators in lymph nodes and tumor sites in addition to changing how biologics interact with target cells [99,100]. Song et al. designed and synthesized albumin nanoparticles, Nano-PI, containing PI3Kγ inhibitors IPI-549 and paclitaxel (PTX), which promoted the delivery and accumulation of these two drugs in lymph nodes and tumor macrophages. Nano-PI combined with anti-programmed death 1 (α-PD1) can promote M2-type repolarization into M1-type macrophages, increase CD4+ and CD8+ T cells, and decrease Tregs cells to reshape the lymph node and tumor immune microenvironment, resulting in the remission of breast cancer in mice [101].

SPNI, a polymer nano immunomodulator, was created by Liu et al. On the one hand, toll-like receptor 7 (TLR7) agonists are released under the stimulation of the acidic tumor microenvironment after reaching the tumor site. On the other hand, SPIN can also provide NIR photodynamic immunotherapy. Additionally, this therapy can aid in both the activation of the immune system and the establishment of immune memory, achieving anti-tumor effects [102]. Pt(IV)/CQ/PFH NPs-^D^PPA-1 is a tumor microenvironment-sensitive nano-ultrasound contrast agent developed by Yang et al. that releases chloroquine, inhibiting cisplatin-induced protective autophagy in 4T1 cells. Pt(IV)/CQ/PFH NPs-^D^PPA-1 promoted cell apoptosis and induced G0/G1 cell cycle arrest and ROS production in vitro. In addition, further studies revealed that Pt(IV)/CQ/PFH NPs-^D^PPA-1 can reprogram the metabolic pathways of immature dendritic cells and TAMs to reverse the tumor immunosuppressive microenvironment, including promoting the differentiation of immature stump cells (iDCs) into mature dendritic cells (mDCs), promoting the polarization of M2-type macrophages into M1-type macrophages, increasing the proportion of CD8+ IFN+ T cells, and promoting the secretion of immune cytokine IL-12. Animal experiments also confirmed that Pt(IV)/CQ/PFH NPs-^D^PPA-1 had anti-breast-cancer and immunosuppressive autophagy activation effects as well as good biocompatibility in vivo [103].

### 3.5. Reversing Drug Resistance

Drug resistance greatly affects the efficacy of tumor treatment. Both chemotherapy drugs and targeted drugs are prone to drug resistance [104,105]. Only a few advanced tumors, including certain leukemia and lymphoma tumors, can be cured with anticancer drugs [106]. However, even these tumors can develop drug resistance. The mechanism of anticancer drug resistance is very complex and includes 1. drug uptake, transport, and activation disorders; 2. increased decomposition, metabolism, and excretion of drugs; 3. increased cell repair mechanisms; and 4. changes in the quality and quantity of targets [29]. It is well known that the activation of the yes-associated protein (YAP) can induce EGFR inhibitor resistance [107]. As a consequence, YAP has also become a potential target for reversing resistance to EGFR inhibitors. Polymer@Gef-YAP-siRNA NPs targeting the co-delivery of gefitinib and YAP-siRNA were created by Huang et al.; they represent a possible therapeutic strategy for NSCLC resistance to EGFR-TKIs. Nanoparticles could efficiently enter gefitinib-resistant NSCLC cells and release gefitinib and YAP-siRNA. In a gefitinib-resistant xenograft model, Polymer@Gef-YAP-siRNA NPs preferentially accumulated at tumor sites, producing a strong anti-tumor effect without obvious toxicity after laser irradiation. Studies showed that the main anti-tumor mechanisms of this therapy were as follows: (1) gefitinib blocked the EGFR signaling pathway; (2) YAP-siRNA inhibited the activation of the EGFR bypass signaling pathway–YAP/MEK/ERK signaling pathway; and (3) photodynamic therapy induced the apoptosis of tumor cells [108].

P-glycoprotein (P-gp) is a transmembrane glycoprotein with an energy-dependent “drug pump” function. P-gp can bind to both medicines and ATP, which provides energy to pump intracellular drugs out of cells, lowering the concentration of intracellular medications and resulting in drug resistance [109,110]. Many researchers have confirmed that P-gp is one of the promising therapeutic targets for overcoming drug resistance in cancer [111,112,113]. A class of anticancer drugs known as P-gp substrates can be encapsulated into nanoparticles to avoid the drug resistance caused by P-gp drug efflux [114]. Wang’s research team developed fucoidan-decorated silica-carbon nano-onion nanoparticles (FSCNO NPs), which not only suppress tumor blood vessels by targeting P-selectin but also release P-gp inhibitors and anticancer drugs under low-power NIR to reverse tumor drug resistance [115]. RIP-SAHA is a novel nano-radiosensitizer for breast cancer. It has good colloidal stability and not only promotes the intracellular release of SAHA in tumor cells to enhance DNA damage and restrain DNA repair but also further enhances the sensitivity of tumor cells to radiotherapy due to the high iodine properties of RIP. In conclusion, RIP-SAHA effectively enhances the radiotherapy effect of breast cancer [116].

**Table 3:** Heterocyclic-associated nanomedicines.

Drug Effect	Drug Name	Mechanism	Reference
Inhibiting tumor cells	Nano-MP@PSI	Targeting MDMX/p53	[61]
	Nano-ERASER	Degradation of the target protein	[62]
	ODDBP-NPs	Targeting PP2A/DNA	[63]
	ECX NVs	NA	[64]
	GS-HA/GS-PTX-HA	NA	[65]
Inhibiting CSCs	iPBCGC NPs	Targeting CD44	[69]
	SFN/M-HA-SS-TA	Targeting CD44	[75]
	HA-NAP-NPs	Targeting CD44/COX-independent pathway	[76]
Inhibiting angiogenesis	V@LDL NPs+ Vandetanib	Targeting VEGF	[86]
	WB@hydrogel	Releasing NO to up-regulate p53 protein	[94]
	PTX-siRNA^VEGF^-NPs	Targeting VEGF	[95]
Regulating the immune microenvironment	Nano-PI + α-PD1	M2-type repolarization into M1-type macrophages; increasing CD4+ and CD8+T cells; decreasing Tregs cells	[101]
	SPNI	NIR photodynamic immunotherapy	[102]
	Pt(IV)/CQ/PFH NPs-^D^PPA-1	Promoting DC cell maturation; M2-type repolarization into M1-type; increasing CD8+ IFN+ T cells and IL-12	[103]
Reversing drug resistance	Polymer@Gef-YAP-siRNA NPs	Targeting EGFR; targeting YAP/MEK/ERK signaling pathway; apoptosis	[108]
	FSCNO NPs	Targeting P-selectin and P-gp	[115]
	RIP-SAHA	Enhancing DNA damage and restraining DNA repair; enhancing radiotherapy sensitivity	[116]

NA: Not applicable.

## 4. Perspective and Conclusions

Targeted therapy, in contrast to conventional chemotherapy, has the benefits of high specificity and minimal side effects, allowing for effective individual treatment of tumors [117]. Small molecule inhibitors are characterized by easy synthesis and modification, good stability, high membrane permeability, and easy penetration of drugs into tumor cells. In addition, they have clear targets and clear mechanisms of action, and specific and effective inhibition of targets reducing drug toxicity and side effects. Heterocyclic stents are an essential component of many anticancer drugs and play a significant role in the creation of new drugs. This review focused on heterocyclic targeted drugs. Heterocyclic targeted drugs can exert anti-tumor effects by acting on a single target or even multiple targets in tumor cells or the TME. The application of nanotechnology in medicine can not only improve the accuracy of tumor diagnosis but also boost the precision of tumor treatment. Nanomaterials can be used as drug carriers to deliver drugs to specific target sites, release drugs, and even realize the simultaneous delivery of multiple drugs to achieve the precise treatment of tumors. Furthermore, we also reviewed heterocyclic-associated nanomedicines in cancer. Heterocyclic-associated nanomedicines can play an anti-tumor role by directly inhibiting tumor cells, tumor stem cells, and tumor angiogenesis, regulating the immune microenvironment, and even reversing drug resistance by packaging drugs and using P-gp inhibitors and upstream signaling inhibitors. However, because the gene regulatory network that controls tumor incidence and progression is so intricate, it is impossible to treat a tumor completely with a single targeted medicine. Additionally, although targeted therapy’s side effects are less severe than those of conventional chemotherapy, these should be closely monitored. Future research on tumor genetics, proteomics, and pharmaceutical technology will necessitate the development of novel “highly selective” targeted medications and nanomedicines with improved clinical outcomes. Moreover, few heterocyclic-associated nanomedicines have entered clinical trials, and even fewer have been approved for clinical application. The future clinical implementation of heterocyclic-associated nanomedicines depends on a careful assessment and resolution of the safety and regulatory issues.

## Data Availability

Not applicable.
